# Effects of 15-Deoxy-Δ^12,14^-Prostaglandin J2 (15d-PGJ2) and Rosiglitazone on Human Vδ2^+^ T Cells

**DOI:** 10.1371/journal.pone.0007726

**Published:** 2009-11-04

**Authors:** Haishan Li, C. David Pauza

**Affiliations:** Institute of Human Virology, University of Maryland School of Medicine, Baltimore, Maryland, United States of America; New York University School of Medicine, United States of America

## Abstract

**Background:**

Thiazolidinediones (TZD) class of drugs, and 15-deoxy-D12,14-prostaglandin J2 (15d-PGJ2) are immune regulators predicted to modulate human autoimmune disease. Their effects on γδ T cells, which are involved in animal model and human and animal autoimmune diseases, are unknown.

**Methodology/Principal Findings:**

We characterized the activity of rosiglitazone (from the TZD class of drugs) and 15d-PGJ2 in human Vδ2 T cells. We found that 15d-PGJ2 and rosiglitazone had different effects on Vδ2 T cell functions. Both 15d-PGJ2 and rosiglitazone suppressed Vδ2 T cell proliferation in response to IPP and IL2. However, only 15d-PGJ2 suppressed functional responses including cytokine production, degranulation and cytotoxicity against tumor cells. The mechanism for 15d-PGJ2 effects on Vδ2 T cells acts through inhibiting Erk activation. In contrast, rosiglitazone did not affect Erk activation but the IL2 signaling pathway, which accounts for rosiglitazone suppression of IL2-dependent, Vδ2 T cell proliferation without affecting TCR-dependent functions. Rosiglitazone and 15d-PGJ2 are designed to be peroxisome proliferator-activated receptor gamma (PPARγ) ligands and PPARγ was expressed in Vδ2 T cell. Surprisingly, when PPARγ levels were lowered by specific siRNA, 15d-PGJ2 and rosiglitazone were still active, suggesting their target of action induces cellular proteins other than PPARγ.

**Conclusions/Significance:**

The current findings expand our understanding of how the immune system is regulated by rosiglitazone and 15d-PGJ2 and will be important to evaluate these compounds as therapeutic agents in human autoimmune disease.

## Introduction

The incidence of autoimmune disease has been growing in recent years and the contribution to disease of various immune cell subsets are being defined. Research in autoimmunity focuses primarily on cells of the adaptive immune system and their roles in disease. Several studies implicated γδ T cells in animal models and human autoimmune diseases including multiple sclerosis (MS) [1], [2], experimental allergic encephalomyelitis (EAE) [3], polymyositis [4], [5], Bechet's disease [6], [7], rheumatoid arthritis (RA) [8], atopic dermatitis (AD) [9] and systemic lupus erythematosus (SLE) [10]. Although the exact role for γδ T cells remains unknown, they possess potent cytotoxic activity, are major sources of cytokines including IFN-γ and TNF-α and produce chemokines involved in recruiting monocyte/macrophages [11], [12], [13]. Recently, γδ T cells in mouse were reported to be an important source of IL17 [14], [15], [16], [17], [18]. These functions of activated γδ T cells could contribute significantly towards inflammatory processes and promote autoimmunity.

In humans, γδ T cells represent 1 to 10% of circulating T cells in blood, with the majority (>80%) expressing a Vγ2Vδ2 (also termed Vγ9Vδ2) TCR (hereafter referred as Vδ2 T cells) [19] that mediates broad reactivity against microbial agents and tumors. Cells in this subset recognize low molecular weight, non-peptidic compounds termed “phosphoantigens,” including isopentyl pyrophosphate (IPP) [20], [21], an intermediate in sterol and isoprenoid biosynthesis. Following stimulation by phosphoantigens, Vδ2 T cells proliferate, release cytokines (particularly IFN-γ and TNF-α) [22], [23] or chemokines [24], [25], and acquire cytotoxic activity against tumor cells [26], [27] or infected cells [28]. In view of the similarity between inflammatory processes in pathogen responses and autoimmune diseases, it is not surprising that Vδ2 T cells might participate in both. Thus, potential treatments for autoimmune diseases may involve modulating human γδ T cell function.

Peroxisome proliferator-activated receptor gamma (PPARγ) is a ligand-dependent transcription factor that was recognized originally as a key regulator of adipocyte function [29], [30]. Recent studies reported that PPARγ are expressed in many immune cells [31]; PPARγ ligands down-regulated dendritic cell [32], NK cell [33], B cell [34]and helper T cell [35], and enhanced regulatory T cell responses [36]. Some of the effects were proved to be PPARγ-independent [33], [34], [36]. Consequently, there have been many studies using PPARγ ligands in animal models of autoimmunity including experimental allergic encephalomyelitis, asthma, arthritis, and colitis [37], [38], [39], [40], [41], [42], [43], [44], [45], [46]. The success of this approach has led to the potential use of PPARγ ligands as therapeutic agents in human autoimmune disease [47], even with the knowledge that these compounds may target molecule other than PPARγ.

15d-PGJ2 and the TZD class of drugs are two types of PPARγ ligands that are studied most often. In the present study, we used synthetic PPARγ ligands from the thiazolidinediones (TZD) class of drugs that are used widely for treating type 2 diabetes because they enhance insulin sensitivity [48] and the endogenous PPARγ ligand cyclopentenone prostaglandin15-deoxy-D12,14-prostaglandin J2 (15d-PGJ2) [49]. We tested their effects on human Vδ2 T cell function as a model for their impact on γδ T cells in autoimmune diseases. We also tested several different TZD class drugs, including rosiglitazone, troglitazone and ciglitazone all with similar results. We uncovered a mechanism for Vδ2^+^ T cell inhibition that surprisingly, was partly independent of PPARγ.

## Results

### Both 15d-PGJ2 and Rosiglitazone Suppressed Vδ2 T Cell Proliferation

**Figure 1:**
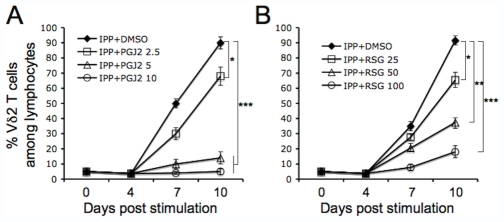
Both 15d-PGJ2 and rosiglitazone suppressed IPP-stimulated Vδ2 T cell proliferation. Fresh isolated PBMC was treated with 15d-PGJ2 (A) or rosiglitazone (B) at various concentrations (µM) for 1 hour, and then stimulated with IPP (15 µM) plus IL2 (100U/ml). Cells were cultured for 10 days by adding IL2 every 3 days. Vδ2 T cell frequency was detected every 3 day. The experiments were set in triplicate. The statistical significance compared with drug vehicle (DMSO) control was analyzed. *, P<0.05; **, P<0.01; ***, P<0.001. Data are representative of at least three independent experiments with different donors.

We tested the effects of PPARγ ligands on Vδ2 T cell proliferative response to phosphoantigen. Freshly isolated PBMC were treated with 15d-PGJ2 or rosiglitazone for 1 hour before adding IPP plus IL2. Cells were cultured for 10 days with IL2 added every 3 days. Vδ2 T cell frequency was measured every 3 days. As shown in figure 1, both 15d-PGJ2 (Figure 1A) and rosiglitazone (Figure 1B) suppressed IPP-driven Vδ2 T cell expansion in a dose-dependent manner. To reach similar effects, a 10-fold higher concentration of rosiglitazone was needed compared to 15d-PGJ2.

### 15d-PGJ2, but not Rosiglitazone, Suppressed Cytokine Production, Degranulation and Cytotoxicity

**Figure 2:**
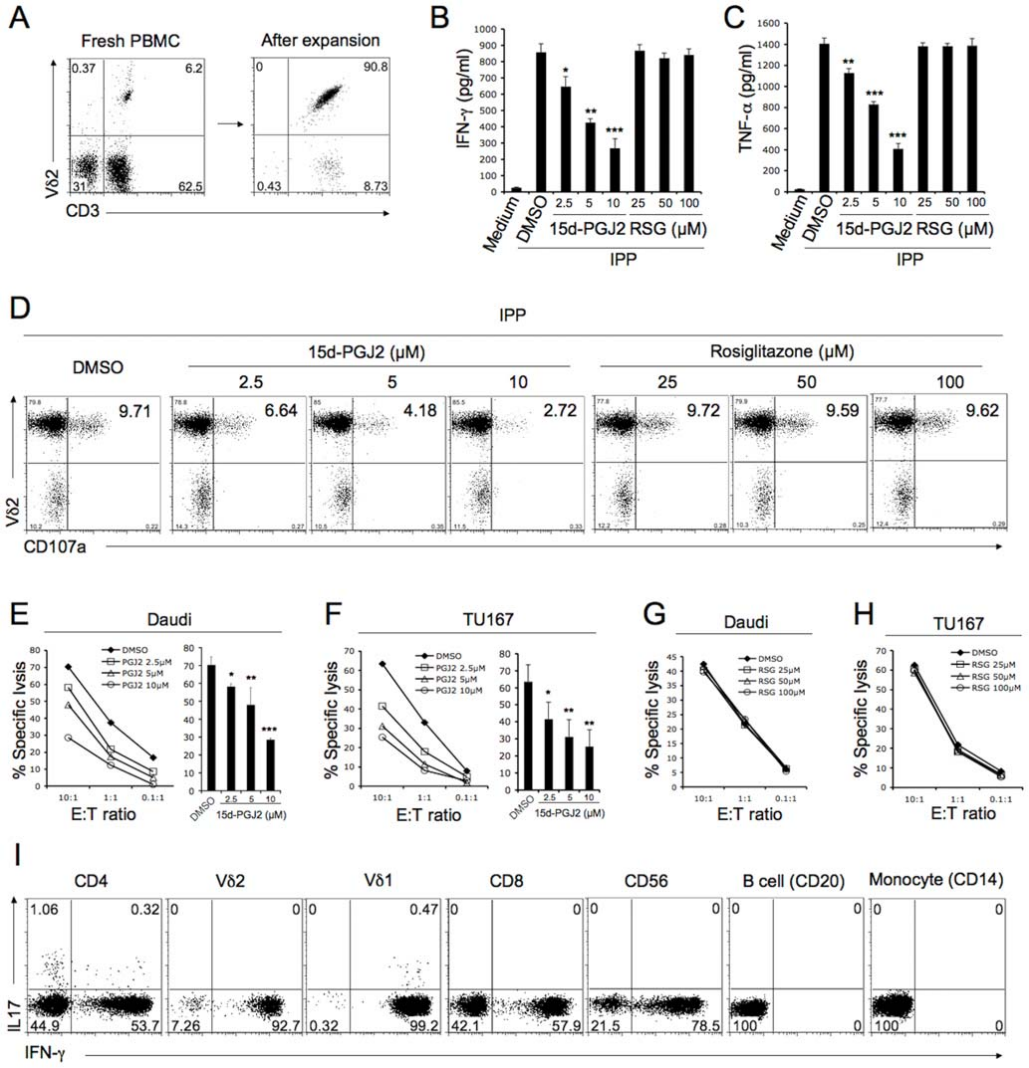
15d-PGJ2, but not rosiglitazone, suppressed cytokine production, degranulation and cytotoxicity functions of Vδ2 T cell. (A) Freshly isolated PBMC contained 1–10% of Vδ2 T cells (left panel); after 10 to 14 days of culture with IPP plus IL-2, the percentage of Vδ2 T cells was more than 90% (right panel). (B, C and D) Vδ2 T cells were treated with 15d-PGJ2 or rosiglitazone at various concentrations for 1 hour and then washed and stimulated with IPP (50 µM). After stimulating for 4 hours, the levels of IFN-γ (B) or TNF-α (C) in cell-free supernatant were detected by antigen capture ELISA. The experiments were done in triplicate and statistical tests compared drug and vehicle (DMSO). CD107a expression (D) was analyzed by flow cytometry. (E and F) Vδ2 T cells were pretreated with 15d-PGJ2 at various concentrations for 1 hour. The cytotoxicity of Vδ2 T cell against Daudi (E) or TU167 (F) was evaluated at different E∶T ratios in triplicate. The statistical significance of specific lysis compared with a drug vehicle (DMSO) control at E∶T = 5∶1 was analyzed. (G and H) Vδ2 T cells were pretreated with rosiglitazone at various concentrations for 1 hour. The cytotoxicity of Vδ2 T cell against Daudi (G) or TU167 (H) was evaluated at different E∶T ratios in triplicate. (I) PBMC was stimulated with PMA (10 ng/ml) and ionomycin (1 µM) for 4 h. IL-17 production in different cell type was detected by flow cytometry. *, P<0.05; **, P<0.01; ***, P<0.001. Data are representative of three independent experiments using different donors.

It was reported previously that expanded Vδ2 T cells (Vδ2 T cell line) but not fresh cells can kill tumor cell targets [27]. Here, we tested whether PPARγ ligands affect phosphoantigen-driven cytokine production and degranulation in Vδ2 T cell lines. Freshly isolated PBMC contained 1–10% of Vδ2 T cells; after 10 to 14 days of culture with IPP plus IL-2, the percentage of Vδ2 T cells was more than 90% (Figure 2A). Vδ2 T cell lines were rested after washing twice and culturing in fresh medium for 24 hours before they were treated with 15d-PGJ2 or rosiglitazone for 1 hour, then washed and stimulated with IPP. There was a dose-dependent suppression by 15d-PGJ2 of Vδ2 T cell IFN-γ (Figure 2B) or TNF-α (Figure 2C) production and degranulation (CD107a expression) (Figure 2D). However, rosiglitazone had no effect on cytokine expression or degranulation, even at very high concentrations (Figure 2B, C and D).

We also tested the effect of PPARγ ligands on Vδ2 T cell line cytotoxicity. Tumor cell lines were Daudi (a Burkitt's lymphoma) and TU167 (a squamous carcinoma). Lysis of both Daudi (Figure 2E) and TU167 (Figure 2F) cells were reduced significantly and in a dose dependent manner when Vδ2 T cells were treated with 15d-PGJ2 before adding to targets. Rosiglitazone did not alter Vδ2 cytotoxicity against Daudi (Figure 2G) or TU167 (Figure 2H) at any of the concentrations tested.

Th17 cells play important roles in autoimmune diseases [50], [51], [52], [53] and a recent study showed that PPARγ selectively inhibits Th17 differentiation [54]. γδ T cells are also recognized as an important source of IL-17 in mice [14], [15], [16], [17], [18]. However, it is not clear whether human γδ T cell can produce IL-17, although a recent study reported that human Vδ1 and Vδ2 T cell from HIV patient can produce both IL-17 and IFN-γ[55]. We stimulated human PBMC from healthy donors with PMA/ionomycin and tested IL-17 production from several immune cell types. The CD4 T cells produced IL-17 as expected, a small frequency of Vδ1 T cell produced both IL-17 and IFN-γ that is consistent with published data [55]. However, we did not find IL-17 production in Vδ2 T cells or other cell types (Figure 2I). We also stimulated Vδ2 T cell with IPP, but we still did not detect IL-17 production (data not shown).

### 15d-PGJ2 Suppressed Vδ2 T Cell Functions by Inhibiting Erk Activation

**Figure 3:**
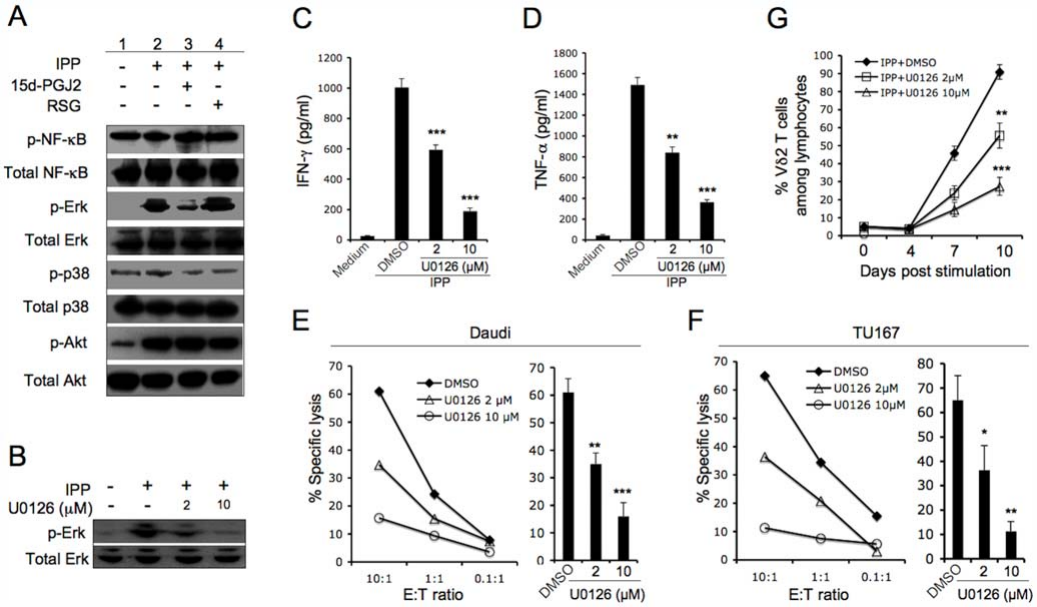
15d-PGJ2 suppressed Vδ2 T cell functions by inhibiting Erk activation. The expanded Vδ2 T cells were rested, incubated in fresh medium for 24 hours without stimulation. (A) The cells were treated with drug vehicle (DMSO), 15d-PGJ2 (10 µM) or rosiglitazone (50 µM) for 1 hour, then stimulated with or without IPP (15 µM). After 30 minutes, cells were collected for western blotting analyses. (B) The cells were treated with drug vehicle (DMSO) or U0126 for 1 hour, then stimulated with or without IPP (15 µM). After 30 minutes, cells were collected for western blotting analyses. (C and D) Vδ2 T cells were treated with drug vehicle (DMSO) or U0126 for 1 hour, then washed and stimulated with IPP. After 4 hours, the levels of cell-free IFN-γ (C) or TNF-α (D) were detected by antigen capture ELISA. The experiments were done in triplicate and statistical tests compared drug with vehicle (DMSO). (E and F) Vδ2 T cells were pretreated with U0126 for 1 hour. The cytotoxicity of Vδ2 T cells against Daudi (E) or TU167 (F) was evaluated at different E∶T ratios. Statistical tests of specific lysis compared drug with vehicle (DMSO) control at E∶T = 5∶1 was analyzed. (G) Fresh isolated PBMC was treated with U0126 at various concentrations for 1 hour, then stimulated with IPP (15 µM) plus IL2 (100 U/ml). Cells were cultured for 10 days by adding IL2 every 3 days. Vδ2 T cell frequencies were detected every 3 days. The experiments were done in triplicate. Statistical tests compared drug with vehicle (DMSO). *, P<0.05; **, P<0.01; ***, P<0.001. Data are representative of three independent experiments with different donors.

The Vδ2 T cell responses to phosphoantigen depends on TCR signaling. We tested 15d-PGJ2 for effects on the Vδ2 TCR signaling pathway as a possible explanation for inhibition of cytokine expression or cytotoxicity. The Vδ2 T cell lines were washed and incubated in fresh medium for 24 hours without stimulation. Then, cells were treated with 15d-PGJ2 or rosiglitazone for 1 hour followed by the addition of IPP. After 30 minutes, cells were collected for western blotting analyses. We measured phosphorylation of several signaling molecules implicated in TCR signal transduction: NFκB, Erk, p38 and PI-3K-associated Akt. Our results demonstrated that NFκB, p38 and Akt were constitutively activated (phosphorylated) in expanded Vδ2 T cell lines, although p38 and Akt were phosphorylated at a lower level compared to NFκB (Figure 3A, lane 1). Phosphorylated Erk was not detected (Figure 3A, lane 1). IPP-stimulation activated Erk and Akt but not NFκB (Figure 3A, lane 2). 15d-PGJ2 but not rosiglitazone suppressed IPP-activated Erk phosphorylation. Neither 15d-PGJ2 nor rosiglitazone affected NFκB, p38, or Akt activation (Figure 3A, lane 3 and 4).

We also tested whether Erk activation is important for Vδ2 T cell function. A highly selective inhibitor of MEK1/2, U0126, was used to inhibit Erk activation. The U0126 inhibited IPP-stimulated Erk activation in Vδ2 T cells in a dose-dependent manner (Figure 3B). When Erk activation was inhibited the functions of Vδ2 T cells, including cytokine production in response to IPP (Figure 3C, D) and cytotoxicity against tumor cells (Figure 3E, F) were suppressed. These results indicated that Erk activation is a key factor in Vδ2 TCR signaling pathway for functional responses. U0126 also suppressed Vδ2 T cell proliferation responses (Figure 3G). Based on these data, we believe that 15d-PGJ2 inhibits Vδ2 T cell functionality by inhibiting Erk activation.

### 15d-PGJ2 and Rosiglitazone Suppressed IL2-Induced Phosphorylation of STAT5 in Vδ2 T Cells

**Figure 4:**
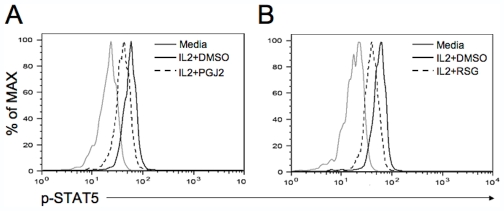
15d-PGJ2 and rosiglitazone suppressed IL2-induced phophorylation of STAT5 in Vδ2 T cells. Purified Vδ2 T cells from fresh PBMC were pretreated with 15d-PGJ2 (10 µM) or rosiglitazone (50 µM) for 1 hour, then incubated with IL2 (100 U/ml) for 15 minutes. The phosphorylated STAT5 was stained with a specific antibody permeabilized cells and detected by flow cytometry.

IPP-driven Vδ2 T cell proliferation depends on IL2. Rosiglitazone inhibited Vδ2 T cell proliferation without affecting the TCR signal. Thus, we postulated that rosiglitazone might inhibit the IL2 signaling pathway. Purified Vδ2 T cells from fresh PBMC were pretreated with 15d-PGJ2 or rosiglitazone for 1 hour, then incubated with IL2 for 15 minutes. The phosphorylated STAT5 was stained with a specific antibody and detected by flow cytometry. Both 15d-PGJ2 (Figure 4A) and rosiglitazone (Figure 4B) suppressed IL2-induced phosphorylation of STAT5 in Vδ2 T cells.

### Primary and Expanded Vδ2 T Cells Express PPARγ

**Figure 5:**
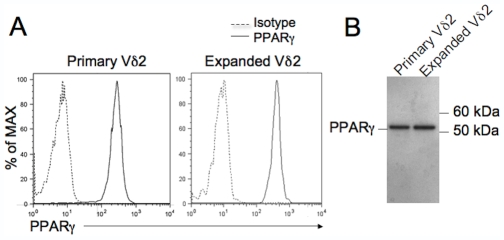
Primary and expanded Vδ2 T cells express PPARγ. The expression of PPARγ in both primary and expanded Vδ2 T cells was examined by flow cytometry using intracellular staining (A) or western blotting (B). Data are representative of two independent experiments.

15d-PGJ2 and rosiglitazone are also PPARγ ligands. We assessed PPARγ expression in primary Vδ2 T cell and IPP-expanded Vδ2 T cell lines. We examined the expression of PPARγ by intracellular staining and flow cytometry. PPARγ was present among Vδ2 cells (Figure 5A). We confirmed the result by western blotting using purified primary or expanded Vδ2 T cells (Figure 5B).

### PPARγ-Independent Effects of 15d-PGJ2 and Rosiglitazone on Vγ2Vδ2 T Cell

**Figure 6:**
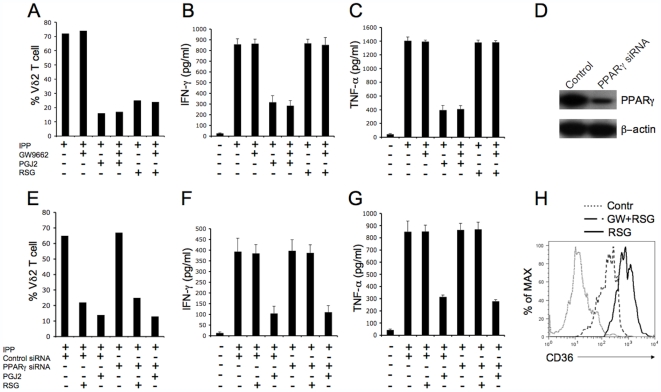
The effects of 15d-PGJ2 and rosiglitazone on Vγ2Vδ2 T cells are PPARγ-independent. (A) Fresh isolated PBMC was pretreated with GW9662 (100nM) for 1 hour, then treated with 15d-PGJ2 (10 µM) or rosiglitazone (50 µM) and cultured with IPP (15 µM) plus IL2 (100 U/ml). Cells were cultured for 10 days by adding IL2 every 3 days. Vδ2 T cell frequencies were detected at day 10. (B and C) The expanded Vδ2 T cells were rested by incubating in fresh medium for 24 hours without stimulation. The cells were pretreated with GW9662 (100 nM) for 1 hour, then washed and treated with 15d-PGJ2 (10 µM) or rosiglitazone (50 µM), and stimulated with IPP. After 4 hours stimulation, the levels of IFN-γ (B) or TNF-α (C) in cell-free supernatants were detected by antigen capture ELISA. (D, E, F and G) Fresh isolated PBMC were transfected with control or PPARγ specific siRNA and cultured for 48 hours. Cells were then collected for western blotting analyses (D); or cells were treated with 15d-PGJ2 (10 µM) or rosiglitazone (50 µM), then cultured with IPP (15 µM) plus IL2 (100 U/ml) for proliferation (E); or Vδ2^+^ T cell were purified and treated with 15d-PGJ2 (10 µM) or rosiglitazone (50 µM) then stimulated with IPP for IFN-γ (F) or TNF-α (G) analyses as described. Data are representative of three independent experiments with different donors. (H) Human monocytes were treated for 16 h with rosiglitazone (10 µM) in the presence or not of GW9662 (100 nM). CD36 expression was analyzed by flow cytometry.

PPARγ ligands have the curious property of acting in both PPARγ-dependent and independent ways. To test whether 15d-PGJ2 or rosiglitazone regulated Vδ2 T cell through PPARγ-dependent or independent mechanism, we used the PPARγ inhibitor GW9662 that covalently modifies the PPARγ ligand-binding domain and acts as an irreversible antagonist at concentrations of 100 nM or less [34], [56]. We used the optimized concentration of 100 nM GW9662 for Vδ2 T cell. As shown in Figure 6, GW9662 alone did not inhibit IPP-driven Vδ2 proliferation (Figure 6A) or cytokine production (Figure 6B, C). Furthermore, GW9662 did not relieve the inhibitory effect of 15d-PGJ2 or rosiglitazone on Vδ2 proliferation (Figure 6A). Also, GW9662 did not prevent the inhibitory effect of 15d-PGJ2 on cytokine production (Figure 6B, C). We next used siRNA to knock down PPARγ and repeated the inhibition studies. A specific siRNA knocked down PPARγ protein levels (Figure 6D) but did not prevent the effects of 15d-PGJ2 or rosiglitazone on Vδ2 T cells (Figure 6E, F and G). These data argue that 15d-PGJ2 and rosiglitazone regulate Vδ2 T cells through PPARγ-independent mechanisms and the molecular target for these drugs has not yet been defined in human γδ T cells. As a positive control, we show here that rosiglitazone increased the expression of CD36 in human monocytes, while GW9662 suppressed the effect of rosiglitazone (Figure 6H), which is consistent with a previous report [57].

## Discussion

In the present study, we report that 15d-PGJ2 and the TZD drug rosiglitazone had different effects on Vδ2 T cell functions. We also elucidated the underlying mechanisms by evaluating signal transduction pathways. This work will be important for understanding the effects of 15d-PGJ2 and the TZD drugs on immune responses and evaluating their application as therapeutic agents in human autoimmune disease.

15d-PGJ2 and rosiglitazone both suppressed Vδ2 T cell proliferation in response to IPP and IL2. Although TZD drugs have a higher binding affinity for PPARγ [31], [48], rosiglitazone was less potent for inhibiting Vδ2 T cell proliferation. Only 15d-PGJ2 suppressed Vδ2 T cell functional responses including cytokine production, degranulation and cytotoxicity against tumor cells. Consequently, the effects of 15d-PGJ2 and rosiglitazone on Vδ2 cell responses to antigen appear to be independent of PPARγ. This hypothesis is supported by recent reports showing 15d-PGJ2 or TZD class drugs act independently of PPARγ. For example, 15d-PGJ2 or TZD drugs modulated regulatory T cell [36], NK cell [33] and astrocyte [58] functions, and induced B cell apoptosis [34] through PPARγ-independent effects. These findings, along with our observation that a specific PPARγ antagonist or siRNA failed to block the effects of 15d-PGJ2 or rosiglitazone in Vδ2 T cells, support the view that a T cell target other than PPARγ is altered by these treatments. The PPARγ was expressed in Vδ2 T cells (Fig. 5) and most immune cells, although non-genomic action of PPARγ ligands have been reported. The continued study of PPARγ in Vδ2 T cell and other immune cell development and function may reveal a yet undiscovered role for PPARγ in the human immune system.

We postulated that 15d-PGJ2 or rosiglitazone might affect the Vδ2 TCR signaling pathway. The αβ TCR signaling pathway has been well studied, but less is known about the γδ TCR and its downstream signaling pathway. A recent paper reported that another phosphoantigen, (E)-4-hydroxy-3-methyl-but-2-enyl pyrophosphate (HMB-PP), induced MEK/Erk- and PI-3K/Akt-mediated signal transduction in primary Vδ2 T cells but IPP failed to induce Akt phosphorylation [59]. In the present study, we tested several key factors in the TCR signaling pathway of Vδ2 T cell lines after IPP stimulation including NFκB, Erk, p38, JNK and Akt. We found that NFκB, p38 and Akt, but not Erk and JNK (data not shown for JNK), were phosphorylated constitutively in Vδ2 T cell lines. IPP-stimulation activated Erk but not JNK, and increased the phosphorylation level of Akt but not NFκB. The NFκB signaling pathway might be important to Vδ2 T cell survival as we reported previously [60]. The Erk and Akt signaling pathway might have greater impact on functional responses by Vδ2 T cells. To test a hypothesis that Erk activation is responsible for Vδ2 T cell function, we inhibited Erk using U0126, a highly selective inhibitor of MEK1/2. We found that the cytokine production and cytotoxicity functions of Vδ2 T cell were suppressed when Erk was inhibited specifically, indicating a key role for Erk in Vδ2 T cell functions. The U0126 also suppressed Vδ2 T cell proliferation, indicating that Erk activation is important for initiating Vδ2 T cell proliferation.

The drugs 15d-PGJ2 and rosiglitazone were distinct in terms of their effects on the Vδ2 TCR signaling pathway. The 15d-PGJ2 but not rosiglitazone specifically inhibited Erk activation. This might explain why only 15d-PGJ2 suppressed Vδ2 T cell effector functions. It was unclear why rosiglitazone suppressed Vδ2 T cell proliferation since it did not affect the Erk activation. Because Vδ2 T cell proliferation requires IL2, we proposed that rosiglitazone might inhibit IL2 signaling. Indeed, both 15d-PGJ2 and rosiglitazone inhibited IL2-induced STAT5 activation, a key factor in the IL2 signaling pathway, arguing that rosiglitazone effects on IL2 signaling explained the inhibition of cell proliferation.

PPARγ ligands are effective in animal models of autoimmunity [38], [39], [41], [42], leading to predictions about therapeutic potential in human disease [47]. The current findings expand our understanding of how the immune system is regulated by PPARγ ligands and will be helpful to evaluate their potential for human therapy. Data presented here also provide important information on the TCR signaling pathway in Vδ2 T cells and will be useful for understanding Vδ2 T cell function during treatments for autoimmune disease.

## Materials and Methods

### PBMC and Tumor Cell Lines

Whole blood was obtained from healthy human volunteers who provided written informed consent and all protocols were approved by the Institutional Review Board at the University of Maryland, Baltimore. Total lymphocytes were separated from heparinized peripheral blood by density gradient centrifugation (Ficoll-Paque; Amersham Biosciences). Peripheral-blood mononuclear cells (PBMC) and TU167 cells (squamous cell carcinoma) were cultured in RPMI 1640 supplemented with 10% fetal bovine serum (FBS; GIBCO), 2 mMol/L L-glutamine, and penicillin–streptomycin (100 U/mL and 100 mg/mL, respectively); for Daudi B cells (CCL-213; ATCC), 4.5 g/L glucose, 1.5 g/L NaHCO_3_, 10 mMol/L HEPES, and 1 mMol/L sodium pyruvate were added.

### In Vitro Proliferation Assays

PBMC (5×10^5^ cells/well) were cultured in 12-well plates with complete medium, 15 µM isopentyl pyrophosphate (IPP) (Sigma) and 100 U/ml human recombinant IL-2 (Tecin, Biological Resources Branch, National Institutes of Health, Bethesda, MD). In some experiments, 15d-PGJ2 or rosiglitazone (Cayman Chemical Company, MI) were added. Fresh complete medium and 100 U/ml IL-2 were added every 3 days. γδ T cell proliferation was measured by staining for CD3 and Vδ2, then defining, by flow cytometry, the percentage of γδ T cells within the total lymphocyte population at days 0, 4, 7 and 10.

### RNA Interference

Fresh isolated PBMC were transfected with control siRNA or a specific siRNA that target PPARγ mRNA (Dharmacon) using a human T cell nucleofactor kit following the manufacturer's instructions (Amaxa Biosystem Inc. USA). Cells were used after 48 hours. The impact of RNA interference was evaluated by immunoblotting for the PPAR-γ protein (see below).

### Purifying Vδ2^+^ Cells and Monocytes

The Vδ2^+^ or CD14^+^ monocyte subsets were purified from fresh PBMC or PBMC expanded with IPP and IL-2 using a MultiSort Kit (MiltenyiBiotec, Auburn, CA) according to the manufacturer's instructions. Cells were stained with PE-conjugate Vδ2 or CD14 antibodies for 10 minutes on ice. The labeled cells were washed and incubated with anti-PE MicroBeads for 15 minutes on ice, then separated in a magnetic field. We achieved 90 to 98% purity after magnetic bead separation as measured by flow cytometry.

### Immunoblot Analysis

Cells (2×10^6^) were lysed in gel loading buffer (Invitrogen, Carlsbad, CA); samples were boiled for 10 minutes and proteins were separated by SDS-PAGE. Proteins were transferred to nitrocellulose membranes and probed with various primary antibodies. Secondary antibodies including HRP-conjugated, anti-rabbit or anti-mouse (Cell Signaling Technology, Inc.) were visualized with enhanced chemiluminescence (GE Healthcare, Buckinghamshire, UK) and exposure to Kodak X-ray film.

### Cytotoxicity Assay

A nonradioactive, fluorometric cytotoxicity assay with calcein-acetoxymethyl (calcein-AM; Molecular Probes) was used to measure cytotoxicity against Daudi B cell or TU167 squamous cell tumor lines [60]. Expanded γδ cells (effector cells) were treated with 15d-PGJ2 or rosiglitazone (Cayman Chemical Company, MI) at varying concentrations for 1 hour at 37°C. Daudi B or TU167 target cells were labeled for 15 minutes with 2 mMol/L calcein-AM at 37°C and then washed once with PBS. Cells were combined at various effector-to-target (E∶T) ratios in 96-well, round-bottomed microtiter plates (Corning, NY) and incubated at 37°C in 5% CO_2_ for 4 hours; assays were performed in triplicate. After incubation, supernatants were transferred to a 96-well flat-bottomed microtiter plate and calcein content was measured using a Wallac Victor2 1420 multi-channel counter (l485/535 nm). Percent specific lysis was calculated as: (test release-spontaneous release)/(maximum release-spontaneous release) ×100.

### Flow Cytometry

Unless noted, cells were stained with fluorophore-conjugated monoclonal antibodies from BD Biosciences, San Jose, CA. Generally, 3×10^5^–5×10^5^ cells were washed, resuspended in 50–100 µL of RPMI 1640, and stained with mouse anti-human Vδ2 -PE clone B6, mouse anti-human CD3-fluorescein isothiocyanate (FITC) clone UCHT1, mouse anti-human CD3-allophycocyanin (APC) clone UCHT1, mouse anti-human CD107a-FITC clone H4A3, and isotype controls, including rabbit anti-mouse IgG1-FITC clone X40, IgG1-PE clone X40, and IgG1-APC clone X40. For detecting intracellular IL-17, cells were stained with mouse anti-human CD4 (PerCP), mouse anti-CD8 (PerCP), mouse anti-human Vδ2-PE, mouse anti-human Vδ1 (FITC), mouse anti-human CD20 (PE), mouse anti-human CD14 (PE), mouse anti-human CD56 (PE), then fixed, permeabilized and incubated for 45 min at 4°C with mouse anti–human IL-17 (FITC or PE). Intracellular staining solutions were obtained from the Cytofix/Cytoperm Kit (BD Biosciences). For intracellular PPARγ and phospho-STAT5 staining, treated and untreated cells were fixed by adding 16% formaldehyde directly into the culture medium to obtain a final concentration of 2∼4% formaldehyde. Cells were incubated in fixative for 10 minutes at 37°C and pelleted. They were then permeabilized by resuspending with vigorous vortexing in 500 µL ice-cold methonal and placed on ice at least 30 minutes. Cells were washed twice in staining medium (PBS containing 0.5% BSA) then resuspended in staining medium at 0.5∼1×10^6^ cells per 100 µL and blocked in staining buffer for 10 minutes at room temperature. Optimal concentrations of mAB against PPARγ (clone E8, Santa Cruz Biotechnology, Inc.) or phospho-STAT5 (pY694, clone 47) were added and incubated for 1 hour at room temperature. Cells were washed with staining buffer and resuspended. Data for at least 1×10^4^ lymphocytes (gated on the basis of forward- and side-scatter profiles) were acquired from each sample on a FACSCalibur flow cytometer (BD Biosciences). All samples were analyzed using FlowJo software (FlowJo 8.8.2, Tree Star, San Carlos, CA). For stimulation before staining, PBMC or Vδ2 T cells were treated with IPP or PMA/ionomycin for 4 hours. In some experiments, Vδ2 T cells were treated with 15d-PGJ2 or rosiglitazone (Cayman Chemical Company, MI) at varying concentrations for 1 hour at 37°C before stimulation and then washed.

### Detecting Cytokines by ELISA

Human IFN-γ in culture supernatants was detected with a human IFN-γ ELISA kit (R&D Systems), according to the manufacturer's directions. Human TNF-α in culture supernatants was detected with a human TNF-α ELISA kit (R&D Systems), according to the manufacturer's directions.

### Statistical Analysis

Differences among groups were analyzed by Student's t test. P<0.05 was considered to be significant.
